# European clinical guidelines for Tourette syndrome and other tic disorders—version 2.0. Part I: assessment

**DOI:** 10.1007/s00787-021-01842-2

**Published:** 2021-10-18

**Authors:** Natalia Szejko, Sally Robinson, Andreas Hartmann, Christos Ganos, Nanette M. Debes, Liselotte Skov, Martina Haas, Renata Rizzo, Jeremy Stern, Alexander Münchau, Virginie Czernecki, Andrea Dietrich, Tara L. Murphy, Davide Martino, Zsanett Tarnok, Tammy Hedderly, Kirsten R. Müller-Vahl, Danielle C. Cath

**Affiliations:** 1 Department of Neurology, Medical University of Warsaw, Warsaw, Poland; 2 Department of Bioethics, Medical University of Warsaw, Warsaw, Poland; 3 Department of Neurology, Yale School of Medicine, Yale University, New Haven, USA; 4 Tic and Neurodevelopmental Movements Service (TANDeM), Children’s Neurosciences Centre, Evelina London Children’s Hospital, Guys and St Thomas’ NHS Foundation Trust, London, UK; 5 Department of Neurology, Salpetriere Hospital, Paris, France; 6 Department of Neurology, Charité Universitätsmedizin Berlin, Berlin, Germany; 7 Paediatric Department, Herlev University Hospital, Herlev, Denmark; 8 Clinic of Psychiatry, Social Psychiatry and Psychotherapy, Hannover Medical School, Hannover, Germany; 9 Department of Clinical and Experimental Medicine, University of Catania, Catania, Italy; 10 Department of Neurology, St George’s Hospital, St George’s University of London, London, UK; 11 Institute of Neurogenetics, University of Lübeck, Lübeck, Germany; 12 Department of Child and Adolescent Psychiatry, University Medical Center Groningen, University of Groningen, Groningen, The Netherlands; 13 Tic Disorder Clinic, Great Ormond Street Hospital NHS Foundation Trust, London, UK; 14 Department of Clinical Neurosciences, University of Calgary, Calgary, Canada; 15 Vadaskert Child Psychiatry Hospital, Budapest, Hungary; 16 Department of Psychiatry, University Medical Center Groningen, Rijks Universiteit Groningen, GGZ Drenthe Mental Health Institution, Hanzeplein 1, Assen, 9713 Groningen, The Netherlands

**Keywords:** Tics, Tourette syndrome, Assessment, Scales

## Abstract

In 2011 a working group of the European Society for the Study of Tourette Syndrome (ESSTS) has developed the first European assessment guidelines for Tourette syndrome (TS). Now, we present an updated version 2.0 of these European clinical guidelines for Tourette syndrome and other tic disorders, part I: assessment. Therefore, the available literature has been thoroughly screened, supplemented with national guidelines across countries and discussions among ESSTS experts. Diagnostic changes between DSM-IV and DSM-5 classifications were taken into account and new information has been added regarding differential diagnoses, with an emphasis on functional movement disorders in both children and adults. Further, recommendations regarding rating scales to evaluate tics, comorbidities, and neuropsychological status are provided. Finally, results from a recently performed survey among ESSTS members on assessment in TS are described. We acknowledge that the Yale Global Tic Severity Scale (YGTSS) is still the gold standard for assessing tics. Recommendations are provided for scales for the assessment of tics and psychiatric comorbidities in patients with TS not only in routine clinical practice, but also in the context of clinical research. Furthermore, assessments supporting the differential diagnosis process are given as well as tests to analyse cognitive abilities, emotional functions and motor skills.

## Introduction

According to DSM-5, tics are defined as sudden, rapid, recurrent, non-rhythmic movements or vocalisations usually appearing in bouts while waxing and waning in frequency, intensity, number, complexity, and kind of tic [1]. Tic disorders including Tourette syndrome (TS) typically first appear in childhood, mostly between age 5 and 6 years [2]. TS encompasses the combination of chronic (more than 1 year) motor and vocal tics. Although the diagnosis of TS is in most cases straightforward, the condition is often under-recognised and many patients receive the correct diagnosis many years after the onset of symptoms. In these cases, delayed access to appropriate treatments both for tics and for commonly occurring neuropsychiatric comorbidities such as obsessive–compulsive disorder (OCD) and attention deficit/hyperactivity disorder (ADHD) may hamper psychosocial development and negatively impact quality of life. Therefore, both early recognition of tics and appropriate assessment of neuropsychiatric comorbidities, even those subclinical, are mandatory steps in the evaluation and treatment of patients with TS [3].

In 2011, experts of the European Society for the Study of Tourette Syndrome (ESSTS) have developed the first European guidelines in four parts [4–7]. Here, we present a revised and updated version of the original 2011 European Guidelines part 1: assessment. With these guidelines, we offer practical aids for clinicians from the neighbouring fields of psychiatry, neurology, paediatrics and psychology on how to assess the various characteristics of patients with tic disorder.

## Methods

For the new version of part I of the European clinical guidelines, we conducted a literature search, primarily aiming at detecting relevant research on the assessment of tics as well as co-existing comorbid conditions, and on quality of life, published after first guidelines between January 2011 and May 2021. Our systematic approach was based on the search in PubMed, Ovid, Web of Science, Embase, and APA Psych Info conducted on March 2020 and again on May 31, 2021. We searched for articles reporting about assessment of tics and TS using the search terms “tics” AND/OR “Tourette” AND/OR “assessment” AND/OR “scales” AND/OR “quality of life” AND/OR “OCD” AND/OR “ADHD” AND/OR “depression” AND/OR “anxiety”. Reviews and meta-analyses in the area were further searched for relevant citations. In addition, the reference lists of the articles identified were reviewed for additional studies. In addition to the studies identified through systematic review, to make the publication list as comprehensive as possible, studies still in press and not officially published were added by the authors (i.e. through precedent knowledge about relevant publications). The methodology of the ESSTS survey is presented in a summary paper in the current issue of this journal Epidemiology of tics and tic-related comorbidities.

### Prevalence

TS affects between 0.3 and 1% of the general population [8–10], depending on the age of the study group and rigorousness of the sampling method used in the research. Tics occur predominantly in young people (before age 18), and typically have a waxing and waning course [11]. Tics occur more often in boys than in girls, with a male to female preponderance of between 3:1 [12] and 4.3:1 [13, 14], but in adult patients with TS this male preponderance is less pronounced [15]. Both genetic and individual environmental factors contribute to the tic/TS phenotype [16–22].

### Course and course prediction of tics and comorbidities

The mean age at onset is around 5 years although lower ages at onset are reported in up to 40% of patients [23]. In children and adolescents, waxing and waning is the rule, whereas, in adults tics tend to run a more persistent course [24]. Complex tics generally present later than simple ones, with vocal tics often appearing 1 or 2 years later than motor tics [25], although in some patients vocal tics appear first [26]. For most patients, the worst period of tics occurs between 8 and 12 years of age [27, 28]. The course of tics is relatively favourable over time. Clinical as well as population-based studies indicate that up to 80% of persons who have presented with a tic disorder before 10 years of age, experience a significant tic decrease during adolescence. By 18 years of age tic intensity and frequency has decreased to such an extent that the majority of people no longer experience significant impairment from tics, although most individuals still have mild tics [29]. Yet, a small proportion of patients does not experience a clinically meaningful decrease in tic intensity, and others continue to experience a severe and debilitating form of tic disorder [25]. Reports on whether certain types of tics in childhood predict tics or comorbidity in adulthood are somewhat conflicting [24, 30–33]. Recently it has been shown that tics, obsessive–compulsive disorder (OCD) and attention deficit/hyperactivity disorder (ADHD) severity in childhood were related to high tic scores, OCD or ADHD diagnoses in early adulthood [34] but longitudinal studies remain rare and replication is needed.

## Diagnosing

### Classifications

Since the original guidelines were published, the fifth revision of the Diagnostic and Statistical Manual of Mental Disorders (DSM-5; APA, 2013) and the eleventh revision of the International Classification of Disease (ICD-11; WHO, 2019) have been published. Both include changes to the classification of tic disorders. Table 1 provides an overview of the differences between DSM-5 and ICD-11 classification. In DSM-5, tic disorders are classified as ‘motor disorders’ within the neurodevelopmental disorders category that also includes intellectual disabilities, communication disorders, autism spectrum disorders (ASD) and ADHD. Within the motor disorders category, tic disorders are grouped alongside developmental coordination disorder and stereotypic movement disorder. The diagnostic categories for tics include Tourette’s disorder (307.21), persistent (chronic) motor or vocal tic disorder (307.22) (with prevalence of 3–9% [35]), provisional tic disorder (307.21), unspecified tic disorder (307.20) and other specified tic disorder (307.20). Importantly, just recently, it has been suggested that all these disorders belong to the same spectrum, TS being the most severe one [36].

**Table 1:** Differences between DSM-IV-TR, DSM-5, ICD-10, ICD-11

	Labels	Parent category	Criteria of TS	Criteria of chronic/persistent vocal and/or motor tic disorder	Criteria of provisional/transient tic disorder
DSM-IV-TR	Tourette’s disorder; chronic motor or vocal tic disorder; transient tic disorder; tic disorder not otherwise specified	Disorders of infancy, childhood, and adolescence	Multiple motor and one or more vocal tics at some point in illness Tics occur daily or periodically, but 1 year since onset, and no tic-free period of more than 3 consecutive months Onset before 18 years Not caused by substance or other condition	One or more motor or vocal tics present at some point, not both motor and vocal symptoms Tics occur daily or periodically, but 1 year since onset, and no tic-free period of more than 3 consecutive months Onset before 18 years Not caused by substance or other condition No history of TS	One or more motor and vocal tics Tics occur daily or periodically, but for 4 weeks and 12 months Onset before 18 years Not caused by substance or other condition No history of TS Specify if single episode or recurrent
ICD-10	Combined vocal and multiple motor tic disorder (de la Tourette); chronic motor or vocal tic disorder; transient tic disorder; other tic disorders; tic disorder, unspecified	Behavioural and emotional disorders with onset usually occurring in childhood and adolescence	Multiple motor and one or more vocal tics, not necessarily occurring at the same time	One or more motor or vocal tics, but not both types Symptoms occur 12 months	One or more motor and/or vocal tics Symptoms occur 12 months
DSM-5	Tourette’s disorder; persistent (chronic) motor or vocal tic disorder provisional tic disorder; other specified tic disorder; unspecified tic disorder	Neurodevelopmental disorders	Multiple motor and one or more vocal tics at some point in illness May wax and wane, but have persisted 1 year since onset Onset before 18 years Not caused by substance or other condition	One or more motor or vocal tics present at some point, not both motor and vocal symptoms May wax and wane, but have persisted 1 year since onset Onset before 18 years Not caused by substance or other condition No history of TS Specify if motor tics only, vocal tics only	One or more motor and/or vocal tics Tics present for 1 year since onset Onset before 18 years Not caused by substance or other condition No history of TS or persistent tic disorder
ICD-11	Tourette syndrome (combined vocal and motor tic disorder); persistent (chronic) motor or phonic tics; provisional tic disorder; substance-induced tic disorder; tic disorder due to general medical condition	Disorders of nervous system—primary; mental and behavioural disorders—secondary; obsessive–compulsive and related disorders; neurodevelopmental disorders	One or more motor and/or vocal tics occurring over the same period of time Symptoms occur 12 months	One or more motor and one or more vocal tics Symptoms occur 12 months	One or more motor or vocal tics, but not both types Symptoms occur 2 weeks and 12 months

For the previous version of this Table, refer to the 2011 ESSTS Guidelines [4]

*DSM-IV-TR* The Diagnostic and Statistical Manual of Mental Disorders Text Revision 4th edition, *DSM-5* The Diagnostic and Statistical Manual of Mental Disorders 5th edition, *ICD-10* the International Statistical Classification of Diseases and Related Health Problems, 10th edition, *ICD-11* the International Statistical Classification of Diseases and Related Health Problems, 11th edition

Compared with previous DSM-IV-TR classifications, the definition of tics has been refined, and the term *stereotyped* to distinguish between stereotypies and tics has been removed. The duration criterion of a tic-free period of less than three consecutive months has been omitted for the chronic tic disorders. Provisional tic disorder replaces transient tic disorder, because a transient nature of tics can only be defined retrospectively and initially presenting tics may eventually be diagnosed as chronic tic disorder. The category of persistent tic disorder has been specified, i.e. at least one vocal or two motor tics should be present, to distinguish between vocal and motor tics that are chronic. The unspecified and other specified tic disorder categories have additionally been introduced to replace tic disorders not otherwise specified, to account for tics with onset in adulthood or tics triggered by other medical conditions or use of medications and drugs. Stimulant use as a specific cause of tics has been removed.

In ICD-11, TS is removed from the category of emotional disorders and classified under the category of movement disorders. In our opinion this is in disregard of the growing body of evidence pointing into the positioning of tics and TS as a psychiatric and emotional disorder (for more details consult the “European clinical guidelines for Tourette Syndrome and other tic disorders. Summary statement” in the current issue of this journal).

### Characteristics of tics

Tic characteristics have been described in detail in the 2011 assessment guidelines and are summarised in Table 2. Because of their importance to the clinical assessment process, here the key points are summarised: (1) tics are either motor or vocal in nature. Motor tics reflect brief, sudden, irresistible, inapposite and non-rhythmic recurrent movements in voluntary muscles or muscle groups. Vocal tics reflect sounds elicited by a flow of air through the vocal cords, mouth or nose; (2) tics are often associated with essential characteristics that distinguish them from other hyperkinetic movement disorders, which include (i) suggestibility by environmental cues, (ii) a preceding premonitory urge or tension, (iii) mostly a feeling of voluntariness when performing the tic, and (iv) the ability of temporary suppression that is often accompanied by an inner tension.

**Table 2:** Different types of tics and their characteristics

Type of tic	Typical features
Motor	Arise in the voluntary musculature and involve discrete muscles or muscle groups
Vocal	Consist of any noise produced by movement of air through the nose, mouth or pharynx
Stimulus-bound	Occur in response to internal or external stimuli (visual, phonic, tactile or mental)
Blocking	Motor or vocal tics that interrupt the voluntary action without alteration of consciousness (dysfluency of speech or gait)
Simple	Are restricted to one muscle or a single muscle group (e.g. eye blinking, nose twitching, tongue protrusion), simple, meaningless sounds (e.g. grunting, throat clearing, coughing, sniffling and barking)
Complex	Involvement of more muscle groups (e.g. repetitive touching of objects or people, repetitive obscene movements (copropraxia), mimicking others (echopraxia) complex vocal tics are words or phrases, expressing obscenities (coprolalia), repeating others (echolalia) or repeating oneself (palilalia))
Clonic	Last less than 100 ms
Dystonic	Last more than 300 ms Repetitively abnormal posture of a kind that one may see in dystonia
Tonic	Last more than 300 ms Relatively long duration of the contraction (in e.g. back muscles) without exhibiting abnormal postures

These features vary across patients with differing levels of suggestibility, premonitory urges and suppressibility that are in part related to age and tic severity [37]. In addition, the same tic burden may be associated with different intensities of premonitory urges [36, 38–40]. The distribution of premonitory urges often co-varies with the distribution of tics [41]. Higher levels of premonitory urges have been reported to be associated with greater awareness of tic expression, with only moderate to low associations between subjective and objective measures of tics [42]. Clinical experience shows that most people with tics are not aware of all of their tics. Most typically, mild simple motor tics such as eye tics may escape patients’ attention. Accordingly, some individuals report that they are “tic-free” despite the presence of mild tics on video recordings [40].

Since the publication of the first version of the ESSTS guidelines [4] there has been also a growing interest in the cognitive and sensory characteristics of tics and the role of attentional processes [43–45].

### Role of genetics in the diagnosis of tic disorders

From family studies it is well-known that (a history of) tics is presented in a substantial proportion of parents [9] and that patients’ first-degree families have a higher risk of developing tics, obsessive–compulsive symptoms (OCS) or ADHD compared to healthy control families [46]. Accordingly, three epidemiological twin- and family studies and two genome-wide association studies (GWAS) have shown heritability estimates ranging between 28 and 56% [47–51], with the remaining variance being explained by unique environmental factors. Interestingly, a recent large GWAS meta-analysis suggested that TS and other primary tic disorders share the same polygenic risk [16].

Genetic complex trait analysis (GCTA) revealed that single-nucleotide polymorphisms (SNPs) with minor allele frequency (MAF) < 5% seem to explain 21% of the variance in TS [52].

Although variants in different TS risk-genes have been identified (e.g. CNTN6, NRXN1, SLITRK1 and HDC), they are responsible for genetic vulnerability only in a very small proportion (about 1%) of TS affected individuals [53]. Other findings from recent GWAS such as a genome-wide significant locus within the brain-expressed gene FLT3 on chromosome 13 could not be replicated [16, 50]. Thus, there is no doubt that in TS, both genetic and environmental factors contribute to the onset and persistence of the disease. Despite that, to date, no specific genetic markers have been identified.

## Tic-related phenomena

The clinical presentation of TS includes a range of tic-related phenomena that have been extensively studied in recent years due to their impact on daily functioning for individuals with TS.

### Compulsive tics/tic-like behaviours and impulsive tics

Complex motor tics often have a compulsive nature and can be indistinguishable from goal directed or OCD-like behaviours, for example when a tic is repeated until a “just-right feeling” is achieved. Common compulsive tics of this nature include repeated touching, tapping and evening-up (e.g. brushing against something on one side and then the other). For some individuals these compulsive tics/tic-like behaviours are performed a fixed number of times and/or aim at neutralising an anxiety-driven worry (often about preventing harm). However, for the majority of individuals they are not anxiety-driven worries, but instead are performed to satisfy a feeling of sensory discomfort with engagement until a ‘just right’ feeling is achieved [54]. Interestingly, the commonly reported “non-just-right”-feelings that accompany symmetry behaviour in TS has a parallel with the premonitory urges preceding tics in TS [55].

### Self-injurious behaviours

Self-injurious behaviours (SIB) are highly associated with complex motor tics and coprophenomena in patients with TS, can occur in response to provocative stimuli in the outside world rather than OCD or OCS [37]. However, self-injuries in patients with TS are not only associated with tics, but also with a number of other co-occurring symptoms. These include hyperactivity and accident-proneness in the scope of ADHD, rage attacks or excessive washing or grooming resulting in skin lesions in patients with co-occurring OCD [56].

## Quality of life in TS

Children and adolescents with tics experience a poorer quality of life than healthy children [57], with poorer quality of life related to increased tic severity [58] and mostly associated with comorbid ADHD and OCD [58–61]. However, their overall quality of life is better than in youth with other psychiatric disorders. Poor quality of life in adults with TS, is associated with the presence of comorbid OCD and depression and to a lesser extent with tic symptom severity [58]. The effect of anxiety and OCS severity on quality of life is often mediated by depression severity [58].

## Comorbidities

In the following paragraph we want to address clinically important and in particular new aspects related to comorbidities in TS.

### OCD and ADHD

Obsessions and compulsions occur in between 22 and 66% of clinical TS populations [23, 62] and are, together with ADHD (occurring in 55–60%) the most prevalent comorbid disorders in patients with TS. Especially the OC symptom dimension of symmetry and non-just-right behaviour is highly prevalent in TS [63], but all other OCD symptom dimensions including checking, ordering and washing occur as well. Interestingly, hoarding behaviour has been found to have genetic overlap both with TS, OCD and ADHD [64]. In patients with TS and comorbid ADHD it can be difficult to differentiate tics from fidgetiness and hyperactivity. However, tics are repetitive and patterned movements that alternate completely normal motor patterns, which clearly distinguish them from overall behavioural hyperactivity and restlessness. Comorbid ADHD is associated with increased rates of OCD, anxiety, anger control as well as personality and mood disorders [62, 65, 66].

### Autism spectrum disorder (ASD)

Only recently attention has been directed towards relationships between TS and ASD, with two studies of TS patients and family members, and one comparative study between TS, ASD and general population subjects [67–69]. All participants completed several quantitative self-reports on autistic personality traits, including subscales on restrictive and repetitive behaviours. Overall, up to 22.8% of children with TS met cut-off criteria for ASD (22.8%), but only 8.7% of the TS adults. The elevated rate in the studies in children was primarily due to high scores on the Social Responsiveness Scale (SRS) Repetitive and Restricted Behaviours (RRB) subscale, which bears striking resemblances with OCD symptoms. Specifically, children with clinicians’ diagnosis of TS plus OCD exhibited elevated SRS scores indicating symptom overlap between assessments of OCD and ASD on this scale. Fully in line with these results, the two studies in TS adults investigating underlying factor structure across scales on tics, OC symptoms, ADHD and autistic symptoms [59, 68] revealed strikingly similar results, with one overlapping factor between OC and autistic symptoms, defined by the numbers and patterns subscale of the autism self-report.

### Rage attacks

Episodic impulse control disturbances and anger control problems (commonly referred to as ‘rage attacks’) are reported in 25–75% of patients with tic disorders. The episodes are described as abrupt outbursts of verbal or physical aggression generally directed at persons in the vicinity of the patient. The episodes are seen as being in excess of the response required of the eliciting stimulus [70]. Rage attacks are often associated with comorbid disorders such as ADHD, ASD, emotional liability, affective dysregulation characteristic for disruptive mood disorder and OCD [71], but also occur in a proportion of patients with “TS only” without any comorbidities. Budman et al. used the newly developed “Rage Attacks Questionnaire” (RAQ) to assess rage attacks in children and adolescents [72]. While they suggested that episodic rage in TS represents a nonspecific symptom, in a recent study in adults using a revised version of this scale (The Rage Attack Questionnaire-Revised, RAQ-R) [73], it could be demonstrated that rage attacks are significantly more common in TS compared to controls and can be clearly differentiated from impulsivity as indicated by a low correlation between the RAQ-R and established rating scales for impulsivity. Although rage attacks occurred more often in individuals with comorbid ADHD, they were also found in patients with “TS only”, independently from comorbid ADHD, impulsivity, and OCD. Rage attacks were found to negatively influence patients’ quality of life [73].

## Neuropsychological impairments

Children with TS are at risk of academic underachievement, grade retention and are in the need for additional support [74], particularly those with more severe tics and co-existing conditions [75]. In addition, the prevalence of tics in children with special educational needs is as high as 28% [76]. Adults with TS can suffer from neurocognitive difficulties, which impact on their daily function [77]. The literature on neurocognitive impairments is somewhat contradictory, as study samples differ with respect to age (children vs adults) and comorbidity patterns, with the consequence of different cognitive profiles [78–80]. Intelligence is generally considered to fall within the average range in individuals with TS. As an exception, a Danish paediatric clinical cohort of children with TS reported lower non-verbal and full-scale intellectual efficiency (less than one standard deviation) in comparison with a control group, which correlated with disease duration and presence of co-occurring conditions [81]. The authors concluded that early onset of tics (and not disease duration) might be associated with specific deficits of cognitive performance. Difficulties with motor skills [82] and visual perceptual abilities [83] are also reported. Specific learning disorders are also known to be frequent in children with TS [75], particularly difficulties with mathematics and handwriting [84–86]. Although the literature is inconsistent, there is some indication that executive dysfunction in TS is specifically related to reduced inhibitory control [87] and cognitive flexibility [88]. Impairments in sustained attention, working memory, habit/procedural learning, and social cognition are also found; deficits are often stronger in the presence of comorbid ADHD or OCD [89–92] (for review see [87]). In adults executive dysfunction has been found in persons with “TS only” even in the absence of co-occurring conditions [93]. Hypothetically, the adult clinical group represents a more severe group of patients with persistent tics.

Interestingly, some studies have found enhanced abilities in TS patients with respect to inhibitory control [82, 94], procedural memory [95], and habit formation [96]. Inhibitory control seems to improve over time and be less disabling in adulthood, in parallel with tic severity decrease [97].

## Differential diagnosis

### Other hyperkinetic movement disorders

The distinction of tics from other hyperkinetic movements (e.g. dystonia, myoclonus and chorea), or vocalisations in the context of neurodegenerative disorders (e.g. repetitive vocalisations) or vocalisations as part of ictal phenomena [98] is in most cases straightforward. However, occasional brief motor tics could be mislabeled as chorea, whereas prolonged motor tics could resemble dystonic contractions. Specifically, eye squeezing as part of blepharospasm can resemble severe eye blinking tics. Most importantly, tics may co-exist with other hyperkinetic movements and in these cases, misdiagnoses are not uncommon [99, 100]. For example, the association of tics with dystonia as a primary syndrome has been previously reported [101].

### Stereotypies

One particular area of confusion in this regard involves stereotypies, which often, as neurodevelopmental phenomena, co-occur with tics. Stereotypies, like tics, are also non-goal directed movement patterns, but are repeated continuously, often quasi-rhythmically [102]. Importantly, stereotypies typically involve more muscle groups than tics (e.g. stereotypic repetitive movements of the trunk and arms/hands, and less common the legs or even come along with vocalisations) and they are most often phenomenologically fixed over longer periods of time. Stereotypies generally start at an earlier age than tics (between age 0–3 years), and, as aforementioned, are often encountered in children with tics or other psychiatric conditions (such as ASD), but may also be part of normal development. Both tics and stereotypies are distractible and suppressible, and the attenuation of stereotypies is less effortful and more instantaneous than tics. Importantly, different from tics, stereotypies are typically not associated with a premonitory urge, but their presence may exert soothing effects, sense of fulfillment or even joy and can come along with fantasy dreams [102–104].

### Functional movement disorders and vocalisations

A challenging differential diagnostic category involves functional tic-like movements and vocalisations. There is a growing literature on functional movement disorders (FMD) and vocalisations, with prevalence rates ranging between 3% in population-based studies and 30% in neurological outpatient clinics [105, 106]. Functional tic-like movements are commonly encountered in specialist clinics for tics and TS. Indeed, over the past few years, several case series have documented the clinical features of these patients [107–111]. The first two reports focussed on a range of atypical features, such as absence of premonitory urges and the inability to voluntarily suppress tics, adult onset of symptoms, female preponderance of patients, as well as differences both in the types of tic movements or sounds and the somatotopy of body parts involved. For example, patients with functional tic-like movements most often exhibit rhythmic complex motor or phonic behaviours, which affect the arms, legs and trunk equally often—or even more often—whereas, in TS face, head or shoulders are primarily affected. Also, typical tics are fast, brief, phasic movements representing fragments of actions. In addition, at a given time, tic repertoire is usually limited and repetitive albeit not rhythmic. Following these first reports, it subsequently became clear that paediatric patients may present with functional tic-like movements as well [112] and indeed it is now recognised that the symptom overlap between patients with tics and functional tic-like movements may be greater than previously thought. Most importantly, both types of behaviours may co-exist in the same patients, although frequencies of co-occurrence are not known. Therefore, in such cases thorough clinical neuropsychiatric assessment in expert centres for tics and TS is warranted. Similarly, functional vocalisations are usually characterised by onset in adulthood, proceeding traumatic events, tendency to block speech and poor response to anti tic medication [111]. Moreover, coprolalia is much more common in functional vocalisations than TS, in which it is present in only 15–20% of patients [113, 114]. Ganos et al. [115] indicate that coprolalic words in patients with functional vocalisations differ from coprolalia in TS. Patients with functional coprolalia use longer and compound words or sentences and included an atypically high number of different swear words including words rarely encountered in TS. In contrast, coprolalia in TS is characterised by usage of short swear words, usually widely known.

Finally, in recent years there has been a growing evidence regarding functional tic-like behaviours induced by social media [116]. Some researchers postulate it could be, at least partially, due to overall increase of mental disorders due to COVID-19 pandemic [116].

### Somatic conditions

Sometimes eye tics are misdiagnosed as ophthalmological conditions, conjunctivitis or dry eye syndrome. Further, patients with predominant vocal tics such as throat clearing or sniffing are sometimes first referred to otolaryngologists, when an allergy is suspected, or to a gastroenterologist to investigate on gastroesophageal reflux. Vocal tics can lead to speech blockade [117] and stuttering-like symptoms that may be misdiagnosed as tonic–clonic stuttering. Motor (or vocal) phenomena resembling tics might be due to focal epileptic activity. In these cases, the abnormal phenomena are characteristically focussed only on specific body parts and their manifestation is stereotypic. Sometimes specific triggers, like for example the presence of photic stimuli, can trigger the abnormal–epileptic–behaviours.

### PANS/PANDAS

The hypothesised causal relationship between tic onset and course with streptococcal infections in children, conceptualised in the concept of Paediatric Acute-Onset Neuropsychiatric Syndrome (PANS) [118, 119]), is a topic of debate. Importantly, a recent large scale collaborative effort across multiple sites in Europe (EMTICS) [119] on the relationship between β-haemolytic Streptococcal infections and tic exacerbation in children and adolescents with TS has indicated no specific relationship [120–122]. Instead, more generally there is substantial evidence for involvement of immunological factors in tic onset and persistence. Moreover, treatment of children with either antibiotics or immune electrophoresis in whom a relationship with streptococcal infections was assumed has not proven efficacy to date [123, 124]. Finally, the phenotypic expression of tic onset as a result of PANS hugely overlaps with general tic onset phenomenology. As a consequence, the use of specific PANS-related scales does not seem to have added value to clarify the diagnostics of TS nor does it have consequences with respect to current treatment approaches. Therefore, we have chosen not to provide specific recommendations with respect to rating scales that measure PANS and Paediatric Autoimmune Neuropsychiatric Disorders Associated with Streptococcal Infections (PANDAS)-related tic symptomatology.

## Work-up

In the context of the updated ESSTS guidelines, we have conducted a survey among the members of the ESSTS working group to investigate, which assessments are most commonly used among specialists in the field (see the “European clinical guidelines for Tourette syndrome and other tic disorders. Summary statement” in the current issue of this journal for a thorough description). In each section of the work-up paragraph hereunder we have included results of the survey.

### General evaluation

A general evaluation of both children and adults includes assessment of the most debilitating complaints and symptoms, assesses how the symptoms have developed and inquiries about potential stressors and triggers. Especially in children and adolescents, a developmental history is obtained, and family functioning is assessed including parental coping styles and parental conflict, social network and financial and housing situation. In adults, partner status, current work and financial/housing situation is assessed as well. Moreover, if available hetero-anamnesis on tics, OCD, ADHD family history and disease status is obtained from parents, partner or caregivers in the vicinity of the patient.

### Physical examination

We recommend physical and particularly neurological examination, when clinically indicated to exclude other neurological diseases in addition to tics. Neuroimaging, EEG and further additional examinations do not add value in establishing the diagnosis of a tic disorder and therefore, should be performed only if clinically indicated. For further information please refer to the previous version of the guidelines [4].

### Parent- and patient rating scales to support the general evaluation

In children, adolescents and adults, it is highly advisable to supplement clinical interviewing with screening of the most prevalent comorbid psychopathology by interviewing parents and children supplemented with self-report scales. For further details see Table 3.

**Table 3:** Tic and comorbidity assessment in children and adults

Topic	Measurement instrument children	Measurement instrument adults	Time (min)
Demographics	Age, sex, education level child and parents, work status parents, ethnicity, child and parents (based on country of origin info), marital status parents	Age, sex, education level, work status, ethnicity patient and parents (based on country of origin info), marital status	Max. 20
Age at onset tics, OCD, ADHD	Age at onset, age at worst ever	Age at onset, age at worst ever	Max. 10
Family history tics/OCD/ADHD	Family tree, including disease in family members	Family tree, including disease in family members	Max. 20
Tic diagnosis according to DSM	Interview (derived from DCI or parts of DISC)	Interview (derived from DCI)	Max. 10
Other DSM diagnoses	Kiddie-SADS-PL	MINI/SCID	Max. 60
Tic symptoms (past/present)	YGTSS	YGTSS	Max. 30
Tic symptoms	STSS	STSS	Max. 30
Tic symptoms	TODS	TODS	Max. 30
Tic symptoms	RVBTRS	RVBTRS	Max. 30
OCD symptoms (past/present)	CY-BOCS, CY-BOCS-II, OCI-CV, LOI-CV, CHOCI	Y-BOCS/D-YBOCS, OCI-R	Max. 30
ADHD	SNAP/CAARS (parent/teacher/self-rating), Qb + © DSM-5 criteria of ADHD [125] ADHD-RS	SNAP/CAARS, Qb + ©, Adult ADHD Self-Report Screening Scale for DSM-5 [126], WURS	Max. 50
Autism symptoms	Social Responsiveness Scale (SRS)	Autism Questionnaire (AQ)	Max. 25
Impulsive behaviour	BIS-15	BIS-15	Max. 5
Sensory premonitory urges	PUTS (10 items) I-PUTS (10 items)	PUTS (10 items) I-PUTS (10 items)	Max. 5 Max. 5
Course of psychopathology			
Severity-tics	YGTSS (11 items; current & worst ever; age at worst ever)	YGTSS (11 items; current & worst ever; age at worst ever)	Max. 15
Severity OC symptoms	CY-BOCS severity (2 9 10 items; current & worst ever)	Y-BOCS severity (2 9 10 items; current & worst ever)	Max. 10
Severity depression and anxiety	RCADS (47 items)	BDI/BAI (42 items)	Max. 20
Psychosocial functioning	CGI, GTS-QOL (28 items)	CGI, GTS-QOL (28 items)	Max. 17
Life events	Brugha (29 items)	Brugha (29 items)	Max. 15
Estimation of patients’ time for the specific baseline measurements	Max. 175 min	Max. 165 min	

*DCI* Diagnostic Confidence Index [127], *DISC* Diagnostic Interview Schedule for Children [128], *Kiddie-SADS-PL* Schedule for Affective Disorders and Schizophrenia for School-Age Children [129], *SCID* Structured Clinical Interview on DSM-5 axis I disorders[130], *MINI* Mini International Neuropsychiatric Interview[131], *CY-BOCS* Children’s Yale-Brown Obsessive Compulsive Scale [132], *CY-BOCS-II* the Children's Yale-Brown Obsessive–Compulsive Scale Second Edition [133], *Y-BOCS* Yale-Brown Obsessive–Compulsive Scale [134], *DY-BOCS* Dimensional Yale-Brown Obsessive–Compulsive Scale [135], *SNAP-IV* Swanson, Nolan and Pelham questionnaire, 4th edition [136], *CAARS* the Connors ADHD Rating Scale, *RS* Social Responsiveness Scale [137], *BIS* Barratt Impulsivity Scale [138], *PUTS* Premonitory Urge Tics Scale [139], *I-PUTS* the Individualised Premonitory Urge for Tics Scale [140], *YGTSS* the Yale Global Tic Severity Scale [141], *RCADS* Revised Child Anxiety and Depression Scale [142], *BDI* Beck Depression Inventory-II [143], *BAI* Beck Anxiety Inventory [144], *CGI* Clinical Global Impression [145], *GTS-QOL* Gilles de la Tourette Syndrome–Quality of Life Scale [146], *Qb* + *©* Quantified Behaviour Test Plus [147], *OCI-CV* Obsessive Compulsive Inventory; child’s version [148], *LOI CV* Leyton Obsessive Inventory Child Version; (in children)/LOI (in adults) [149], *WURS* the Wender Utah Rating Scale [150], *STSS Shapiro* Tourette-Syndrome Severity *Scale *[151]*, TODS* Tourette’s Disorder Scale [152], *RVBTRS* Rush Video-Based Tic Rating Scale [153]

## Specific evaluation

### Clinical interview

Age of onset of first motor and vocal tics are recorded as well as tic history, course and age at worst tic severity. Further, inquiries are made about which tics (or comorbid conditions) are considered to be most debilitating, and about their physical consequences (including pain/injuries of muscles and joints), about somatosensory phenomena accompanying the tics (including character, location, and duration), tic suppressibility (including duration) and about exacerbating or relieving factors accompanying the tics (e.g. stress sensitivity) as well as specific complex tics including copro-, echo- and paliphenomena. Patients and parents are asked about the daily, weekly and monthly course of tic activity (including during sleep), to anticipate future treatment effect in relation to the patients’ natural symptom fluctuations, and to clarify the psychosocial impact of tics on family functioning, learning and quality of life [146], and tic exacerbation.

The clinical examination is accompanied by standardised assessment of tics and comorbid conditions (including ADHD, OCD, self-injurious and anger control behaviours, mood and anxiety, sleep and learning difficulties).

### Assessment of tics

A considerable difficulty in assessing and quantifying tics is caused by (1) the spontaneous variations of tics in an individual over time, (2) the large variability in impact of a given level of tic severity on an individual and their family and (3) the tendency of patients to suppress their tics, especially when in the office with the clinician. Therefore, it is advisable when assessing tics, to use multi-informant data, and to combine direct observation (both at home and in the school/work environment), historical information and—if available—to collect video data [154] particularly of “tic attacks” or other exacerbations with potentially functional components. In particular, in those patients, who do not exhibit any tic during the consultation, video recordings can be very helpful. Moreover, mobile applications such as TicTimer [155] help to measure tic in more objective and comparable way.

Our recommendations on assessments for tics and comorbidities in patients with TS are mainly based on (i) our previous guidelines [4], (ii) a systematic review published in 2017 by the Movement Disorder Society based on experts opinion classifying tic rating scales as “recommended”, “suggested” or “listed” [151] and (iii) a survey conducted among European and American ESSTS members in 2019 asking about current use of rating scales for tics.

In brief, the Movement Disorder Society recommends five scales for the assessment of tics, including severity, impairment and premonitory urges: the Yale Global Tic Severity Scale (YGTSS) [141], the Tourette Syndrome Clinical Global Impression (TS-CGI) [156], the Tourette’s Disorder Scale (TDS) [152], the Shapiro Tourette syndrome Severity Scale (STSSS) [157], and the Premonitory Urges for Tics Scale (PUTS) [139] and the Premonitory Urges for Tic Disorders Scale-Revised (PUTS-R) [158]. Six other scales were rated as “suggested”: the Rush Video-Based Tic Rating Scale (RVTRS) [153], the Motor tic, Obsessions and compulsions, Vocal tic Evaluation Survey (the MOVES) [159], the Tourette Syndrome Global Scale (TSGS) [160], the Global Tic Rating Scale (GTRS) [151], the Parent Tic Questionnaire (PTQ) [161], and the Tourette Syndrome Symptom List (TSSL) [151]. Finally, two screening instruments on both tics and comorbidities, i.e. the Motor tic, Obsession and compulsions, Vocal tic Evaluation Survey and Autism-Tics (MOVES) [159] and the Autism-Tics, ADHD and other Comorbidities inventory (A-TAC) [162], were upgraded to the status of “recommended” while two other instruments (Apter 4-questions screening and Proxy Report Questionnaire for Parents and Teachers) were “suggested.”

### Interviews to establish the diagnosis

To establish the diagnosis of TS, the diagnostic criteria of the newest DSM-5 Tourette’s disorder classification are used. According to the ESSTS survey, 94.3% of respondents use primarily an unstructured clinical interview to diagnose TS in clinical practice.

### Ratings of tic severity

In addition, according to the recent ESSTS survey, 73.6% of clinicians use self-reported and/or interview-derived rating scales to support the diagnosis of a primary tic disorder and to list characteristics and severity of tics. Of these users, all clinicians used the YGTSS, which combines both the assessment of tics and impairment. Taking into account psychometric studies, based on the amount of research conduct on psychometric of the YGTSS, it can also be concluded that the YGTSS has the best evidence for assessing tic severity [163]. According to our survey, clinicians use the YGTSS both in daily clinical practice, as well as in research. In 2018, a revised version of the YGTSS (YGTSS-R) was presented based on a relatively large scaled psychometric study in children and adults [163]. Although the YGTSS showed excellent internal consistency and other psychometric properties, changes in anchor points were proposed for several items of the scale without changing the anchor point descriptions, to reduce the skewness in reporting of some of the items of the YGTSS. To give an example, the authors proposed new tic frequency description which is divided in five new categories: none, minimal, mild, moderate, marked and severe. Moreover, minimal frequency, which is equivalent to rare in the original YGTSS, is equivalent to tics present on a daily basis, which was not the case for the previous YGTSS edition. Importantly, Haas et al. [164] demonstrated acceptable psychometric quality of the YGTSS.

Other frequently used scales in clinical practice and research are self-assessments (Adult Tic Questionnaire (ATQ) [165], TSSL [151] and video assessments (Rush Video-Based Tic Rating Scale [153]). The scales recommended, suggested and reasonable as well as their description are summarised in Table 5.

Taken together, we recommend the YGTSS-R to measure tic severity [141]. Alternatively, the following instruments may be used: Shapiro Tourette-Syndrome Severity Scale (STSS) [151], the Tourette's Syndrome Clinical Global Impression Scale (TS-CGI) [151] and the Tourette Disorder Scale (TODS) [152].

### Assessment of quality of life in TS

In children and in adults, it is paramount not only to assess the degree of impairment, but also the overall quality of life. Loss of quality of life entails that the disorder is time consuming, causes significant distress and interferes with major domains of daily life, such as school, work status and (social) relationships. Quality of life can be reliably measured with various instruments. We recommend the TS-specific quality of life scales [for adults: the Gilles de la Tourette Quality of Life Scale (GTS-QOL) and its equivalent used in paediatric population: the Gilles de la Tourette Syndrome-Quality of Life Scale for children and adolescents (C&A-GTS-QOL)] [146, 170]. Storch et al. [125, 126] developed another scale to measure functional impairment in group of children with tics, the Mini-Child Tourette Syndrome Impairment Scale. Also more general quality of life scales can be implemented, including for example the Short Form Health Survey [36-item from (SF-36) or 12-item version (SF-12)] and EuroQol-5 Dimension [171, 172] which are validated in general and psychiatric populations. An overview and suggestions are given in Tables 4 and 5.

**Table 4:** Impairment assessments in children and adults with tics

Scale	Clinical usefulness	Age Group recommended	Rater	Range of scores	Time (min)
Impairment measured with the Yale Global Tic Severity Scale (YGTSS) [141]	Separately rates impairment due to motor or vocal tics	Adults and children	Health professional and the patient	0–50 (0 is the best score)	Max. 5
Global Assessment of Functioning (C-GAS) [166]	Axis five of DSM-IV TR (2002 [1]) and DSM-5, functioning in all areas of development, school/work and psychosocial functioning	Adults and children	Health professional and the patient	0–90 (90 is the best score)	Max. 10
Clinical Global Severity Scale (CGI-S) [145]	Assesses change in global daily functioning	Adults and children	Health professional	0 = much deteriorated and, via 3 = no change, to 6 = very much improved	Max. 5
The Gilles de la Tourette Syndrome-Quality of Life Scale GTS-QOL [167]	27 item scale is based on the health-related quality of life scale (HR-QOL), contains four domains: psychological problems, cognitive problems, physical/activity of daily living problems and obsessive–compulsive themes	Adults	Self-rating	Response ranges between 0 and 4, range 0–100 (0 is the best score)	Max. 30
The Gilles de la Tourette Syndrome-Quality of Life Scale for Children and Adolescents (C&A-GTS-QOL) [146]	A 27-item scale consisting of 4 subscales (psychological, physical, obsessive–compulsive and cognitive)	Children	Two age-adjusted versions: (1) an interview to be administered by a qualified clinician for children aged 6–12 years and (2) a self-report questionnaire for adolescents aged 13–18 years	Response ranges between 0 and 4, range 0–100 (0 is the best score)	Max. 30
The Mini Child Tourette’s Syndrome Impairment Scale (CTIM) [126]	37-item parent rated instrument covering school, home, and social activities that may be impaired by tics or comorbid problems (including OCD symptoms, depression, anxiety, oppositional/disruptive behaviour, hyperactivity, and inattention)	Children	Parent also self-evaluation and short version are available [168, 169]	Each item is rated as: not at all (0), just a little (1), pretty much (2), or very much (3) problematic for the child due to tics or non-tic symptoms. Range 0–111 separately for tics and non-tics (0 is the best score)	Max. 30

**Table 5:** Scales “recommended”, “suggested” and “reasonable” for evaluation of tics, premonitory urges, impairment and psychiatric comorbidities. Results are based on the ESSTS Survey and recommendations by Martino et al. [151]

Domain of assessment	Scale	Recommendation (in clinical practice or research)
Tics	YGTSS	Recommended
	PTQ/ATQ	Suggested
	TSSL	Suggested
	STSS	Reasonable
	TS-CGI	Reasonable
	CGI-S	Reasonable
	TODS	Reasonable
	RVBTRS	Reasonable
	TSGS	Reasonable
	GTRS	Reasonable
	MOVES rater	Reasonable
	MOVES patient	Reasonable
	PRQPT	Reasonable
	Apter 4-q	Reasonable
Tics and comorbidities	A-TAC	Reasonable
Premonitory urges	PUTS	Recommended
Impairment	Impairment in YGTSS	Recommended
	GTS-QOL	Recommended
	C&A-GTS-QOL	Recommended
	CTIM	Suggested
	C-GAS	Suggested
OCD	Y-BOCS	Recommended
	CY-BOCS	Recommended
ADHD	SNAP	Recommended
	CAARS	Recommended
	WURS	Suggested
	Qb + ©	Reasonable

*YGTSS* the Yale Global Tic Severity Scale, *STSS *Shapiro Tourette-Syndrome Severity Scale,* TS-CGI *Tourette Syndrome-Clinical Global Impression,* TODS *the Tourette’s Disorder Scale,* PUTS *Premonitory Urge for Tics Scale,* RVBTRS* Rush Video-Based Tic Rating Scale, *TSGS* Tourette Syndrome Global Scale, *GTRS* Global Tic Rating Scale, *MOVES* Motor tic, Obsessions and compulsions, Vocal tic Evaluation Survey, *PTQ* Parent Tic Questionnaire, *ATQ* Adult Tic Questionnaire, *TSSL* Tourette Syndrome Symptom List, *A-TAC* Autism–Tics, ADHD, and other Co-morbidities inventory, *PRQPT* Proxy Report Questionnaire for Parents and Teachers, *Apter 4-q* Apter 4-questions screening, *GTS-QOL* The Gilles de la Tourette Syndrome-Quality of Life Scale, *C&A-GTS-QOL* Gilles de la Tourette Syndrome-Quality of Life Scale in children and adolescents, *CTIM* Child Tourette’s Syndrome Impairment Scale, *Y-BOCS* Yale-Brown Obsessive Compulsive Scale, *CY-BOCS* Children’s Yale-Brown Obsessive Compulsive Scale, *SNAP* Swanson, Nolan and Pelham questionnaire, *CAARS* Children’s version of the Connors ADHD Rating Scale, *WURS* the Wender Utah Rating Scale, *Qb* + *©* Quantified Behaviour Test Plus, *C-GAS* The Children's Global Assessment Scale, *CGI-S* The Clinical Global Impression—Severity scale, *ADHD* attention deficit hyperactivity disorder, *OCD* obsessive–compulsive disorder

### Assessment of comorbidities

#### ADHD

A clinical interview supplemented with objective self-administrative and clinically-guided ADHD questionnaires is the most commonly used assessment for diagnosing ADHD in children and adults. In children, parents/caregivers and schoolteachers are the main providers of information about psychosocial factors, with standardised measures used to assess ADHD symptoms and functional impairment compared to children of a similar age. The particular challenge in assessment of adults lies in the gathering of reliable information on behaviour that has started before age 12 to establish an ADHD diagnosis. This can be extremely difficult, particularly if no informants (parents, older siblings or other family members) are available to provide information on childhood behaviour, and when current comorbid depressive or other psychiatric symptoms hamper reliable information provided by the patient.

According to the ESSTS survey, most experts use a combination of both clinical interview and scales to diagnose ADHD, most commonly: Swanson, Nolan and Pelham questionnaire (SNAP) [136], Children’s or adults’ versions of the Connors ADHD Rating Scale (CAARS), the Conners Comprehensive Behaviour Rating Scale (CBRS) [173] and the Wender Utah Rating Scale (WURS) [150]. For summary of available scales as well as recommendations please consult Table 3 and previous version of the guidelines [4].

### Other comorbidities: OCD, anxiety, mood, rage attacks

Assessment of other comorbidities is generally performed using clinical interview supplemented with rating scales, both in diagnosing and differential diagnosis, evaluation of treatment and in the context of clinical trials, as reported by the majority of experts in the ESSTS survey. Assessment scales useful in evaluation of these symptoms in children and adults are summarised in Table 3 and our recommendations are shown in Table 5.

The most widely investigated, used and therefore recommended scale is the Yale-Brown Obsessive–Compulsive Scale (Y-BOCS) and its equivalent used for children: the Children’s Yale-Brown Obsessive Compulsive Scale (CY-BOCS) and its revised version the CY-BOCS-II [133].

Since rage attacks have been identified as a common and disabling symptom not only in children, but also in adults, just recently, a new tool has been developed to specifically asses rage attacks in adults with TS, the RAQ-R [73].

## Assessment of neuropsychological functioning

Contrary to assumptions, it is unlikely that cognitive difficulties solely result from chronic tics and are often associated with co-existing conditions, particularly attentional problems or ADHD and likely also OCD [79, 92], with some evidence for inherent executive function problems (especially inhibition control) in TS [87]. Therefore, only in selected cases, in which patients present with cognitive complaints (such as attention or memory problems), learning/school problems or daily life problems, and particularly in those with comorbid conditions, formal neuropsychological testing is recommended, which may help guide intervention and give recommendations how to support the child at school and the adult in their everyday professional or vocational life. Given the wide difference in accessing experienced neuropsychologists across centres around the world, specific neuropsychological batteries are not advocated, but in Table 6 we summarise well-known and commonly used measures.

**Table 6:** Suggested neurocognitive assessments for use in children and adults with TS

Neuropsychological domains	Children	Adults
Intellectual function	WPPSI–IV WISC-V	WAIS-IV
Attention		
Sustained attention	CPT	CPT
Selective attention	TEA-Ch–II	TAP
Working memory auditory/spatial	Digit span, Corsi blocks	Digit span, Corsi blocks, WMS-IV
Executive functions–cognitive aspects		
Conceptual elaboration/categorization Planning Flexibility Inhibition	D-KEFS BADS-C Rey CFT Stroop GNG Wisconsin CST Trail making A,B	D-KEFS BADS Rey CFT Wisconsin CST Stroop GNG Trail making A,B
Executive functions–behavioural/emotional aspects		
Behavioural inhibition	BRIEF–2	BRIEF-A
Emotional regulation	BADS-C	BADS
Social cognition	Facial expression recognition	Faux-pas test, facial expression recognition, FEEST, SEA
Memory	RVDLT, AVLT	MEM-IV
Visual spatial skills	Benton Test	VOSP, BJLO
Literacy and numeracy	WIAT-III	BDAE (BNT) & CAB-DC
Motor skills and coordination	VMI–6	PP

*AVLT* Auditory Verbal Learning Test [174], *BADS* Behavioural Assessment of the Dysexecutive Syndrome [175], *BADS-C* Behavioural Assessment of the Dysexecutive Syndrome for Children [175], *BDAE* Boston Diagnostic Aphasia Examination [176], *BJLO* Benton Judgement of Line Orientation [177], *BNT* Boston Naming Test [178], *BRIEF*-*2* Behaviour Rating Inventory of Executive Function-Second edition [179], *BRIEF-A* Behaviour Rating Inventory of Executive Function for Adults [180], *CAB-DC* Cognitive Assessment Battery for Dyscalculia, *CPT-III* Continuous Performance Test-Connors Third Edition [181], *D-KEFS* Delis Kaplan Executive Function System [182], *FEEST* Facial Expression of Emotions [183], *GNG* Go no go task [184], *PP* Purdue Pegboard [185], *RVDLT* Rey Visual Design Learning Test [186], *Rey CFT* Rey complex Figure Test [187], *TEA-ch*-2 Test of Everyday Attention in Children-Second edition [188], *TAP* Test of Attentional Performance [188], *VOSP* Visual Object and Space Perception [189], *VMI-6* Beery Buktenika Test of Visual Motor Integration-Sixth edition [190], *WAIS-IV* Wechsler Adult Intelligence Scale-Fourth Edition [191], *Wisconsin CST* Wisconsin Card Sorting Test [192], *WIAT-III* Wechsler Individual Achievement Test-third edition [193], *WPPSI-4* Wechsler Preschool and Primary Scale of Intelligence-Fourth edition [194], *WISC-V* Wechsler Intelligence Scale for Children-Fifth Edition [195], *WMS-IV* Wechsler Memory Scale [196], *SEA* Social Cognition and Emotional Assessment

## Conclusions

TS represents a wide range of tics and co-existing symptoms with a varied and heterogeneous presentation. In this updated guideline, we recommend clinically useful assessments and investigations to capture the tic/TS phenotype, taking developmental issues into account. In our opinion, it is highly advisable to choose instruments that cover the whole age range between infancy and adulthood, so that the time course of symptoms across ages and life stages can adequately be captured. In most situations, a standard interview with a few additional questionnaires and rating scales is sufficient to guide diagnosis and treatment. However, psychiatric comorbidities occur in more than three quarters of patients that may require referral for specialised care. Further, in a minority of cases a more extensive neurological and psychiatric screen is necessary to differentiate tics from other hyperkinetic or psychiatric disorders including functional “tic-like” movements. Finally, neuropsychological assessment can be useful to identify specific learning and cognitive impairments to aid academic progress and reasonable adjustments in life.

## Data Availability

Not applicable.

## References

[1] Regier DA, Kuhl EA, Kupfer DJ (2013). The DSM-5: classification and criteria changes. World Psychiatry.

[2] Neuner I, Ludolph A (2009). Tics and Tourette's syndrome throughout the life span. Nervenarzt.

[3] Mol Debes NM, Hjalgrim H, Skov L (2008). Limited knowledge of Tourette syndrome causes delay in diagnosis. Neuropediatrics.

[4] Cath DC, Hedderly T, Ludolph AG, Stern JS, Murphy T, Hartmann A (2011). European clinical guidelines for Tourette syndrome and other tic disorders. Part I: assessment. Eur Child Adolesc Psychiatry.

[5] Roessner V, Plessen KJ, Rothenberger A, Ludolph AG, Rizzo R, Skov L (2011). European clinical guidelines for Tourette syndrome and other tic disorders. Part II: pharmacological treatment. Eur Child Adolesc Psychiatry.

[6] Verdellen C, van de Griendt J, Hartmann A, Murphy T (2011). European clinical guidelines for Tourette syndrome and other tic disorders. Part III: behavioural and psychosocial interventions. Eur Child Adolesc Psychiatry.

[7] Müller-Vahl KR, Cath DC, Cavanna AE, Dehning S, Porta M, Robertson MM (2011). European clinical guidelines for Tourette syndrome and other tic disorders. Part IV: deep brain stimulation. Eur Child Adolesc Psychiatry.

[8] Knight T, Steeves T, Day L, Lowerison M, Jette N, Pringsheim T (2012). Prevalence of tic disorders: a systematic review and meta-analysis. Pediatr Neurol.

[9] Scharf JM, Miller LL, Gauvin CA, Alabiso J, Mathews CA, Ben-Shlomo Y (2015). Population prevalence of Tourette syndrome: a systematic review and meta-analysis. Mov Disord.

[10] Levine JLS, Szejko N, Bloch MH (2019). Meta-analysis: adulthood prevalence of Tourette syndrome. Prog Neuropsychopharmacol Biol Psychiatry.

[11] Robertson MM, Eapen V, Cavanna AE (2009). The international prevalence, epidemiology, and clinical phenomenology of Tourette syndrome: a cross-cultural perspective. J Psychosom Res.

[12] Centers for Disease Control and Prevention (DHHS/PHS) inMMWR v58 n21 (2007) Prevalence of diagnosed tourette syndrome in persons aged 6-17 years–United States, pp 581–585. Web site: http://www.cdc.gov

[13] Apter A, Pauls DL, Bleich A, Zohar AH, Kron S, Ratzoni G (1993). An epidemiologic study of Gilles de la Tourette's syndrome in Israel. Arch Gen Psychiatry.

[14] Robertson MM (2008). The prevalence and epidemiology of Gilles de la Tourette syndrome. Part 1: the epidemiological and prevalence studies. J Psychosom Res.

[15] Yang J, Hirsch L, Martino D, Jette N, Roberts J, Pringsheim T (2016). The prevalence of diagnosed tourette syndrome in Canada: a national population-based study. Mov Disord.

[16] Yu D, Sul JH, Tsetsos F, Nawaz MS, Huang AY, Zelaya I (2019). Interrogating the genetic determinants of Tourette’s syndrome and other tic disorders through genome-wide association studies. Am J Psychiatry.

[17] Willsey AJ, Fernandez TV, Yu D, King RA, Dietrich A, Xing J (2017). De novo coding variants are strongly associated with Tourette disorder. Neuron.

[18] Abdulkadir M, Mathews CA, Scharf JM, Yu D, Tischfield JA, Heiman GA (2019). Polygenic risk scores derived from a Tourette syndrome genome-wide association study predict presence of tics in the avon longitudinal study of parents and children cohort. Biol Psychiatry.

[19] Buse J, Kirschbaum C, Leckman JF, Münchau A, Roessner V (2014). The modulating role of stress in the onset and course of Tourette's syndrome: a review. Behav Modif.

[20] Brander G, Rydell M, Kuja-Halkola R, Fernández de la Cruz L, Lichtenstein P, Serlachius E (2018). Perinatal risk factors in Tourette's and chronic tic disorders: a total population sibling comparison study. Mol Psychiatry.

[21] Hoekstra PJ, Dietrich A, Edwards MJ, Elamin I, Martino D (2013). Environmental factors in Tourette syndrome. Neurosci Biobehav Rev.

[22] Köhler-Forsberg O, Petersen L, Gasse C, Mortensen PB, Dalsgaard S, Yolken RH (2019). A Nationwide Study in Denmark of the association between treated infections and the subsequent risk of treated mental disorders in children and adolescents. JAMA Psychiat.

[23] Hirschtritt ME, Lee PC, Pauls DL, Dion Y, Grados MA, Illmann C (2015). Lifetime prevalence, age of risk, and genetic relationships of comorbid psychiatric disorders in Tourette syndrome. JAMA Psychiat.

[24] Bloch MH, Leckman JF (2009). Clinical course of Tourette syndrome. J Psychosom Res.

[25] Bloch MH (2013). Clinical course and adult outcome in Tourette syndrome.

[26] Singer HS (2019). Tics and Tourette syndrome. Continuum (Minneap Minn).

[27] Luo F, Leckman JF, Katsovich L, Findley D, Grantz H, Tucker DM (2004). Prospective longitudinal study of children with tic disorders and/or obsessive-compulsive disorder: relationship of symptom exacerbations to newly acquired streptococcal infections. Pediatrics.

[28] Leckman JF, Zhang H, Vitale A, Lahnin F, Lynch K, Bondi C (1998). Course of tic severity in Tourette syndrome: the first two decades. Pediatrics.

[29] Pappert EJ, Goetz CG, Louis ED, Blasucci L, Leurgans S (2003). Objective assessments of longitudinal outcome in Gilles de la Tourette's syndrome. Neurology.

[30] de Groot CM, Bornstein RA, Spetie L, Burriss B (1994). The course of tics in Tourette syndrome: a 5-year follow-up study. Ann Clin Psychiatry.

[31] Coffey BJ, Biederman J, Geller DA, Spencer TJ, Kim GS, Bellordre CA (2000). Distinguishing illness severity from tic severity in children and adolescents with Tourette's disorder. J Am Acad Child Adolesc Psychiatry.

[32] Coffey BJ, Biederman J, Geller D, Frazier J, Spencer T, Doyle R (2004). Reexamining Tic persistence and Tic-associated impairment in Tourette's Disorder: findings from a naturalistic follow-up study. J Nerv Ment Dis.

[33] Bloch MH, Peterson BS, Scahill L, Otka J, Katsovich L, Zhang H (2006). Adulthood outcome of tic and obsessive-compulsive symptom severity in children with Tourette syndrome. Arch Pediatr Adolesc Med.

[34] Groth C, Skov L, Lange T, Debes NM (2019). Predictors of the clinical course of Tourette syndrome: a longitudinal study. J Child Neurol.

[35] Mahjani B, Dellenvall K, Grahnat AS, Karlsson G, Tuuliainen A, Reichert J (2020). Cohort profile: epidemiology and genetics of obsessive-compulsive disorder and chronic tic disorders in Sweden (EGOS). Soc Psychiatry Psychiatr Epidemiol.

[36] Eddy CM, Cavanna AE (2014). Premonitory urges in adults with complicated and uncomplicated Tourette syndrome. Behav Modif.

[37] Sambrani T, Jakubovski E, Müller-Vahl KR (2016). New insights into clinical characteristics of Gilles de la Tourette syndrome: findings in 1032 patients from a single German center. Front Neurosci.

[38] Crossley E, Cavanna AE (2013). Sensory phenomena: clinical correlates and impact on quality of life in adult patients with Tourette syndrome. Psychiatry Res.

[39] Reese HE, Scahill L, Peterson AL, Crowe K, Woods DW, Piacentini J (2014). The premonitory urge to tic: measurement, characteristics, and correlates in older adolescents and adults. Behav Ther.

[40] Müller-Vahl KR, Riemann L, Bokemeyer S (2014). Tourette patients' misbelief of a tic rebound is due to overall difficulties in reliable tic rating. J Psychosom Res.

[41] Ganos C, Hummel FC (2011). My urge, my tic—a missing link between urges and tic inhibition. Cogn Neurosci.

[42] Barnea M, Benaroya-Milshtein N, Gilboa-Sechtman E, Woods DW, Piacentini J, Fennig S (2016). Subjective versus objective measures of tic severity in Tourette syndrome—the influence of environment. Psychiatry Res.

[43] Misirlisoy E, Brandt V, Ganos C, Tübing J, Münchau A, Haggard P (2015). The relation between attention and tic generation in Tourette syndrome. Neuropsychology.

[44] Brandt VC, Lynn MT, Obst M, Brass M, Münchau A (2015). Visual feedback of own tics increases tic frequency in patients with Tourette's syndrome. Cogn Neurosci.

[45] Herrmann K, Sprenger A, Baumung L, Alvarez-Fischer D, Muenchau A, Brandt V (2019). Help or hurt? How attention modulates tics under different conditions. Cortex.

[46] Hanna PA, Janjua FN, Contant CF, Jankovic J (1999). Bilineal transmission in Tourette syndrome. Neurology.

[47] Yu D, Sul JH, Tsetsos F, Nawaz MS, Huang AY, Zelaya I (2019). Interrogating the genetic determinants of Tourette's syndrome and other tic disorders through genome-wide association studies. Am J Psychiatry.

[48] Pinto R, Monzani B, Leckman JF, Rück C, Serlachius E, Lichtenstein P (2016). Understanding the covariation of tics, attention-deficit/hyperactivity, and obsessive-compulsive symptoms: a population-based adult twin study. Am J Med Genet B Neuropsychiatr Genet.

[49] Zilhão NR, Olthof MC, Smit DJ, Cath DC, Ligthart L, Mathews CA (2017). Heritability of tic disorders: a twin-family study. Psychol Med.

[50] Scharf JM, Yu D, Mathews CA, Neale BM, Stewart SE, Fagerness JA (2013). Genome-wide association study of Tourette's syndrome. Mol Psychiatry.

[51] Mataix-Cols D, Isomura K, Pérez-Vigil A, Chang Z, Rück C, Larsson KJ (2015). Familial risks of Tourette syndrome and chronic tic disorders. A population-based cohort study. JAMA Psychiat.

[52] Davis LK, Yu D, Keenan CL, Gamazon ER, Konkashbaev AI, Derks EM (2013). Partitioning the heritability of Tourette syndrome and obsessive compulsive disorder reveals differences in genetic architecture. PLoS Genet.

[53] Huang AY, Yu D, Davis LK, Sul JH, Tsetsos F, Ramensky V (2017). Rare copy number variants in NRXN1 and CNTN6 increase risk for Tourette syndrome. Neuron.

[54] Martino D, Ganos C, Pringsheim TM (2017). Tourette syndrome and chronic tic disorders: the clinical spectrum beyond tics. Int Rev Neurobiol.

[55] Fibbe LA, Cath DC, van den Heuvel OA, Veltman DJ, Tijssen MA, van Balkom AJ (2012). Relationship between movement disorders and obsessive-compulsive disorder: beyond the obsessive-compulsive-tic phenotype. A systematic review. J Neurol Neurosurg Psychiatry.

[56] Szejko N, Jakubczyk A, Janik P (2019). Prevalence and clinical correlates of self-harm behaviors in Gilles de la Tourette syndrome. Front Psychiatry.

[57] Storch EA, Merlo LJ, Lack C, Milsom VA, Geffken GR, Goodman WK (2007). Quality of life in youth with Tourette's syndrome and chronic tic disorder. J Clin Child Adolesc Psychol.

[58] Conelea CA, Woods DW, Zinner SH, Budman C, Murphy T, Scahill LD (2011). Exploring the impact of chronic tic disorders on youth: results from the Tourette Syndrome Impact Survey. Child Psychiatry Hum Dev.

[59] Huisman-van Dijk HM, Matthijssen SJMA, Stockmann RTS, Fritz AV, Cath DC (2019). Effects of comorbidity on Tourette's tic severity and quality of life. Acta Neurol Scand.

[60] Erbilgin Gün S, Kilincaslan A (2019). Quality of life among children and adolescents with Tourette disorder and comorbid ADHD: a clinical controlled study. J Atten Disord.

[61] Liu S, Zheng L, Zheng X, Zhang X, Yi M, Ma X (2017). The subjective quality of life in young people with Tourette syndrome in China. J Atten Disord.

[62] Freeman RD, Fast DK, Burd L, Kerbeshian J, Robertson MM, Sandor P (2000). An international perspective on Tourette syndrome: selected findings from 3,500 individuals in 22 countries. Dev Med Child Neurol.

[63] Worbe Y, Mallet L, Golmard JL, Béhar C, Durif F, Jalenques I (2010). Repetitive behaviours in patients with Gilles de la Tourette syndrome: tics, compulsions, or both?. PLoS ONE.

[64] Hirschtritt ME, Darrow SM, Illmann C, Osiecki L, Grados M, Sandor P (2018). Genetic and phenotypic overlap of specific obsessive–compulsive and attention-deficit/hyperactive subtypes with Tourette syndrome. Psychol Med.

[65] Ganos C, Martino D (2015). Tics and Tourette syndrome. Neurol Clin.

[66] Roessner V, Becker A, Banaschewski T, Rothenberger A (2007). Psychopathological profile in children with chronic tic disorder and co-existing ADHD: additive effects. J Abnorm Child Psychol.

[67] Darrow SM, Grados M, Sandor P, Hirschtritt ME, Illmann C, Osiecki L (2017). Autism spectrum symptoms in a Tourette's disorder sample. J Am Acad Child Adolesc Psychiatry.

[68] Eapen V, McPherson S, Karlov L, Nicholls L, Črnčec R, Mulligan A (2019). Social communication deficits and restricted repetitive behavior symptoms in Tourette syndrome. Neuropsychiatr Dis Treat.

[69] Huisman-van Dijk HM, Schoot RVD, Rijkeboer MM, Mathews CA, Cath DC (2016). The relationship between tics, OC, ADHD and autism symptoms: a cross-disorder symptom analysis in Gilles de la Tourette syndrome patients and family-members. Psychiatry Res.

[70] Chen K, Budman CL, Diego Herrera L, Witkin JE, Weiss NT, Lowe TL (2013). Prevalence and clinical correlates of explosive outbursts in Tourette syndrome. Psychiatry Res.

[71] Kumar A, Trescher W, Byler D (2016). Tourette syndrome and comorbid neuropsychiatric conditions. Curr Dev Disord Rep.

[72] Budman CL, Rockmore L, Stokes J, Sossin M (2003). Clinical phenomenology of episodic rage in children with Tourette syndrome. J Psychosom Res.

[73] Müller-Vahl KR, Kayser L, Pisarenko A, Haas M, Psathakis N, Palm L (2020). The Rage Attack Questionnaire-Revised (RAQ-R): assessing rage attacks in adults with Tourette syndrome. Front Psychiatry.

[74] Claussen AH, Bitsko RH, Holbrook JR, Bloomfield J, Giordano K (2018). Impact of Tourette syndrome on school measures in a nationally representative sample. J Dev Behav Pediatr.

[75] Burd L, Freeman RD, Klug MG, Kerbeshian J (2005). Tourette syndrome and learning disabilities. BMC Pediatr.

[76] Stefl ME, Rubin M (1985). Tourette syndrome in the classroom: special problems, special needs. J Sch Health.

[77] Evans J, Seri S, Cavanna AE (2016). The effects of Gilles de la Tourette syndrome and other chronic tic disorders on quality of life across the lifespan: a systematic review. Eur Child Adolesc Psychiatry.

[78] Eddy CM, Cavanna AE (2017). Set-shifting deficits: a possible neurocognitive endophenotype for Tourette syndrome without ADHD. J Atten Disord.

[79] Morand-Beaulieu S, Leclerc JB, Valois P, Lavoie ME, O'Connor KP, Gauthier B (2017). A review of the neuropsychological dimensions of Tourette syndrome. Brain Sci.

[80] Bornstein RA (1990). Neuropsychological performance in children with Tourette's syndrome. Psychiatry Res.

[81] Debes NM, Lange T, Jessen TL, Hjalgrim H, Skov L (2011). Performance on Wechsler intelligence scales in children with Tourette syndrome. Eur J Paediatr Neurol.

[82] Kalsi N, Tambelli R, Aceto P, Lai C (2015). Are motor skills and motor inhibitions impaired in Tourette syndrome? A Review. J Exp Neurosci.

[83] Khalifa N, Dalan M, Rydell AM (2010). Tourette syndrome in the general child population: cognitive functioning and self- perception. Nord J Psychiatry.

[84] Como PG (2001). Neuropsychological function in Tourette syndrome. Adv Neurol.

[85] Mitchell JW, Cavanna AE (2013). Handwriting abnormality in Tourette syndrome. J Neuropsychiatry Clin Neurosci.

[86] Zanaboni Dina C, Bona AR, Zekaj E, Servello D, Porta M (2016). Handwriting tics in Tourette’s syndrome: a single center study. Front Psychiatry.

[87] Morand-Beaulieu S, Grot S, Lavoie J, Leclerc JB, Luck D, Lavoie ME (2017). The puzzling question of inhibitory control in Tourette syndrome: a meta-analysis. Neurosci Biobehav Rev.

[88] Lange F, Seer C, Müller-Vahl K, Kopp B (2017). Cognitive flexibility and its electrophysiological correlates in Gilles de la Tourette syndrome. Dev Cogn Neurosci.

[89] Sukhodolsky DG, Landeros-Weisenberger A, Scahill L, Leckman JF, Schultz RT (2010). Neuropsychological functioning in children with Tourette syndrome with and without attention-deficit/hyperactivity disorder. J Am Acad Child Adolesc Psychiatry.

[90] Eddy CM, Cavanna AE (2013). Altered social cognition in Tourette syndrome: nature and implications. Behav Neurol.

[91] Brandt VC, Moczydlowski A, Jonas M, Boelmans K, Bäumer T, Brass M (2019). Imitation inhibition in children with Tourette syndrome. J Neuropsychol.

[92] Openneer TJC, Forde NJ, Akkermans SEA, Naaijen J, Buitelaar JK, Hoekstra PJ (2020). Executive function in children with Tourette syndrome and attention-deficit/hyperactivity disorder: cross-disorder or unique impairments?. Cortex.

[93] Eddy CM, Rickards HE, Cavanna AE (2012). Executive functions in uncomplicated Tourette syndrome. Psychiatry Res.

[94] Jackson GM, Draper A, Dyke K, Pépés SE, Jackson SR (2015). Inhibition, disinhibition, and the control of action in Tourette syndrome. Trends Cogn Sci.

[95] Takács Á, Kóbor A, Chezan J, Éltető N, Tárnok Z, Nemeth D (2018). Is procedural memory enhanced in Tourette syndrome? Evidence from a sequence learning task. Cortex.

[96] Delorme C, Salvador A, Valabrègue R, Roze E, Palminteri S, Vidailhet M (2016). Enhanced habit formation in Gilles de la Tourette syndrome. Brain.

[97] Yaniv A, Benaroya-Milshtein N, Steinberg T, Ruhrman D, Apter A, Lavidor M (2018). Executive control development in Tourette syndrome and its role in tic reduction. Psychiatry Res.

[98] Mainka T, Balint B, Gövert F, Kurvits L, van Riesen C, Kühn AA (2019). The spectrum of involuntary vocalizations in humans: a video atlas. Mov Disord.

[99] Ganos C, Mencacci N, Gardiner A, Erro R, Batla A, Houlden H (2014). Paroxysmal kinesigenic dyskinesia may be misdiagnosed in co-occurring Gilles de la Tourette Syndrome. Mov Disord Clin Pract.

[100] Ganos C, Münchau A, Bhatia KP (2014). The semiology of tics, Tourette's, and their associations. Mov Disord Clin Pract.

[101] Damásio J, Edwards MJ, Alonso-Canovas A, Schwingenschuh P, Kägi G, Bhatia KP (2011). The clinical syndrome of primary tic disorder associated with dystonia: a large clinical series and a review of the literature. Mov Disord.

[102] Martino D, Hedderly T (2019). Tics and stereotypies: a comparative clinical review. Parkinsonism Relat Disord.

[103] Singer HS (2009). Motor stereotypies. Semin Pediatr Neurol.

[104] Specht MW, Mahone EM, Kline T, Waranch R, Brabson L, Thompson CB (2017). Efficacy of parent-delivered behavioral therapy for primary complex motor stereotypies. Dev Med Child Neurol.

[105] Factor SA, Podskalny GD, Molho ES (1995). Psychogenic movement disorders: frequency, clinical profile, and characteristics. J Neurol Neurosurg Psychiatry.

[106] Stone J, Carson A, Duncan R, Roberts R, Warlow C, Hibberd C (2010). Who is referred to neurology clinics?—the diagnoses made in 3781 new patients. Clin Neurol Neurosurg.

[107] Baizabal-Carvallo JF, Jankovic J (2017). Functional (psychogenic) stereotypies. J Neurol.

[108] Demartini B, Ricciardi L, Parees I, Ganos C, Bhatia KP, Edwards MJ (2015). A positive diagnosis of functional (psychogenic) tics. Eur J Neurol.

[109] Janik P, Milanowski L, Szejko N (2014). Psychogenic tics: clinical characteristics and prevalence. Psychiatr Pol.

[110] Ganos C, Martino D, Espay AJ, Lang AE, Bhatia KP, Edwards MJ (2019). Tics and functional tic-like movements: can we tell them apart?. Neurology.

[111] Ganos C, Edwards MJ, Muller-Vahl K (2016). "I swear it is Tourette's!": on functional coprolalia and other tic-like vocalizations. Psychiatry Res.

[112] Robinson S, Hedderly T (2016). Novel psychological formulation and treatment of “Tic Attacks” in Tourette syndrome. Front Pediatr.

[113] Senberg A, Münchau A, Münte T, Beste C, Roessner V (2021). Swearing and coprophenomena—a multidimensional approach. Neurosci Biobehav Rev.

[114] Kobierska M, Sitek M, Gocyła K, Janik P (2014). Coprolalia and copropraxia in patients with Gilles de la Tourette syndrome. Neurol Neurochir Pol.

[115] Ganos C, Edwards MJ, Müller-Vahl K (2016). “I swear it is Tourette's!”: on functional coprolalia and other tic-like vocalizations. Psychiatry Res.

[116] Heyman I, Liang H, Hedderly T (2021). COVID-19 related increase in childhood tics and tic-like attacks. Arch Dis Child.

[117] Ganos C, Müller-Vahl K, Bhatia KP (2015). Blocking phenomena in Gilles de la Tourette Syndrome. Mov Disord Clin Pract.

[118] Chiarello F, Spitoni S, Hollander E, Matucci Cerinic M, Pallanti S (2017). An expert opinion on PANDAS/PANS: highlights and controversies. Int J Psychiatry Clin Pract.

[119] Thienemann M, Murphy T, Leckman J, Shaw R, Williams K, Kapphahn C (2017). Clinical management of pediatric acute-onset neuropsychiatric syndrome: part I-psychiatric and behavioral interventions. J Child Adolesc Psychopharmacol.

[120] Martino D, Schrag A, Anastasiou Z, Apter A, Benaroya-Milstein N, Buttiglione M (2021). Association of Group A Streptococcus exposure and exacerbations of chronic tic disorders: a multinational prospective cohort study. Neurology.

[121] Cavanna AE, Coffman KA (2021). Streptococcus and tics: another brick in the wall?. Neurology.

[122] Baglioni V, Coutinho E, Menassa DA, Giannoccaro MP, Jacobson L, Buttiglione M (2019). Antibodies to neuronal surface proteins in Tourette syndrome: lack of evidence in a European paediatric cohort. Brain Behav Immun.

[123] Zykov VP, Shcherbina AY, Novikova EB, Shvabrina TV (2009). Neuroimmune aspects of the pathogenesis of Tourette’s syndrome and experience in the use of immunoglobulins in children. Neurosci Behav Physiol.

[124] Hoekstra PJ, Minderaa RB, Kallenberg CG (2004). Lack of effect of intravenous immunoglobulins on tics: a double-blind placebo-controlled study. J Clin Psychiatry.

[125] Garris JF, Huddleston DA, Jackson HS, Horn PS, Gilbert DL (2021). Implementation of the mini-child Tourette syndrome impairment scale: relationships to symptom severity and treatment decisions. J Child Neurol.

[126] Storch EA, Lack CW, Simons LE, Goodman WK, Murphy TK, Geffken GR (2007). A measure of functional impairment in youth with Tourette's syndrome. J Pediatr Psychol.

[127] Robertson MM, Banerjee S, Kurlan R, Cohen DJ, Leckman JF, McMahon W (1999). The Tourette syndrome diagnostic confidence index: development and clinical associations. Neurology.

[128] Lewin AB, Mink JW, Bitsko RH, Holbrook JR, Parker-Athill EC, Hanks C (2014). Utility of the diagnostic interview schedule for children for assessing Tourette syndrome in children. J Child Adolesc Psychopharmacol.

[129] Kaufman J, Birmaher B, Brent D, Rao U, Flynn C, Moreci P (1997). Schedule for affective disorders and schizophrenia for school-age children-present and lifetime version (K-SADS-PL): initial reliability and validity data. J Am Acad Child Adolesc Psychiatry.

[130] Lobbestael J, Leurgans M, Arntz A (2011). Inter-rater reliability of the structured clinical interview for DSM-IV Axis I disorders (SCID I) and Axis II disorders (SCID II). Clin Psychol Psychother.

[131] Sheehan DV, Sheehan KH, Shytle RD, Janavs J, Bannon Y, Rogers JE (2010). Reliability and validity of the Mini International Neuropsychiatric Interview for Children and Adolescents (MINI-KID). J Clin Psychiatry.

[132] Scahill L, Riddle MA, McSwiggin-Hardin M, Ort SI, King RA, Goodman WK (1997). Children's Yale-Brown Obsessive Compulsive Scale: reliability and validity. J Am Acad Child Adolesc Psychiatry.

[133] Storch EA, McGuire JF, Wu MS, Hamblin R, McIngvale E, Cepeda SL (2019). Development and psychometric evaluation of the children's yale-brown obsessive-compulsive scale second edition. J Am Acad Child Adolesc Psychiatry.

[134] Goodman WK, Price LH, Rasmussen SA, Mazure C, Fleischmann RL, Hill CL (1989). The Yale-Brown Obsessive Compulsive Scale. I. Development, use, and reliability. Arch Gen Psychiatry.

[135] Storch EA, Shapira NA, Dimoulas E, Geffken GR, Murphy TK, Goodman WK (2005). Yale-Brown Obsessive Compulsive Scale: the dimensional structure revisited. Depress Anxiety.

[136] Bussing R, Fernandez M, Harwood M, Wei H, Garvan CW, Eyberg SM (2008). Parent and teacher SNAP-IV ratings of attention deficit hyperactivity disorder symptoms: psychometric properties and normative ratings from a school district sample. Assessment.

[137] Constantino JN, Davis SA, Todd RD, Schindler MK, Gross MM, Brophy SL (2003). Validation of a brief quantitative measure of autistic traits: comparison of the social responsiveness scale with the autism diagnostic interview-revised. J Autism Dev Disord.

[138] Patton JH, Stanford MS, Barratt ES (1995). Factor structure of the Barratt impulsiveness scale. J Clin Psychol.

[139] Woods DW, Piacentini J, Himle MB, Chang S (2005). Premonitory Urge for Tics Scale (PUTS): initial psychometric results and examination of the premonitory urge phenomenon in youths with Tic disorders. J Dev Behav Pediatr.

[140] McGuire JF, McBride N, Piacentini J, Johnco C, Lewin AB, Murphy TK (2016). The premonitory urge revisited: an individualized premonitory urge for tics scale. J Psychiatr Res.

[141] Leckman JF, Riddle MA, Hardin MT, Ort SI, Swartz KL, Stevenson J (1989). The Yale Global Tic Severity Scale: initial testing of a clinician-rated scale of tic severity. J Am Acad Child Adolesc Psychiatry.

[142] Chorpita BF, Yim L, Moffitt C, Umemoto LA, Francis SE (2000). Assessment of symptoms of DSM-IV anxiety and depression in children: a revised child anxiety and depression scale. Behav Res Ther.

[143] Beck AT, Ward CH, Mendelson M, Mock J, Erbaugh J (1961). An inventory for measuring depression. Arch Gen Psychiatry.

[144] Beck AT, Epstein N, Brown G, Steer RA (1988). An inventory for measuring clinical anxiety: psychometric properties. J Consult Clin Psychol.

[145] Busner J, Targum SD (2007). The clinical global impressions scale: applying a research tool in clinical practice. Psychiatry (Edgmont).

[146] Cavanna AE, Luoni C, Selvini C, Blangiardo R, Eddy CM, Silvestri PR (2013). The Gilles de la Tourette Syndrome-Quality of Life Scale for children and adolescents (C&A-GTS-QOL): development and validation of the Italian version. Behav Neurol.

[147] Hirsch O, Christiansen H (2016). Factorial structure and validity of the quantified behavior test plus (Qb+©). Assessment.

[148] Foa EB, Coles M, Huppert JD, Pasupuleti RV, Franklin ME, March J (2010). Development and validation of a child version of the obsessive compulsive inventory. Behav Ther.

[149] Berg CJ, Rapoport JL, Flament M (1986). The Leyton obsessional inventory-child version. J Am Acad Child Psychiatry.

[150] Ward MF, Wender PH, Reimherr FW (1993). The Wender Utah Rating Scale: an aid in the retrospective diagnosis of childhood attention deficit hyperactivity disorder. Am J Psychiatry.

[151] Martino D, Pringsheim TM, Cavanna AE, Colosimo C, Hartmann A, Leckman JF (2017). Systematic review of severity scales and screening instruments for tics: critique and recommendations. Mov Disord.

[152] Shytle RD, Silver AA, Sheehan KH, Wilkinson BJ, Newman M, Sanberg PR (2003). The Tourette's Disorder Scale (TODS): development, reliability, and validity. Assessment.

[153] Goetz CG, Pappert EJ, Louis ED, Raman R, Leurgans S (1999). Advantages of a modified scoring method for the Rush Video-Based Tic Rating Scale. Mov Disord.

[154] Goetz CG, Leurgans S, Chmura TA (2001). Home alone: methods to maximize tic expression for objective videotape assessments in Gilles de la Tourette syndrome. Mov Disord.

[155] Black JK, Koller JM, Black KJ (2021). TicTimer Web: software for measuring tic suppression remotely. F1000Research.

[156] Cohen SC, Leckman JF, Bloch MH (2013). Clinical assessment of Tourette syndrome and tic disorders. Neurosci Biobehav Rev.

[157] Shapiro AK, Shapiro ES, Young JG, Feinberg TE (1988). Gilles de la Tourette syndrome.

[158] Baumung L, Müller-Vahl K, Dyke K, Jackson G, Jackson S, Golm D (2021). Developing the premonitory urges for tic disorders scale-revised (PUTS-R). J Neuropsychol.

[159] Gaffney G, Sieg K, Hellings J (1994). The MOVES: a self-rating scale for Tourette's syndrome. J Child Adolesc Psychopharmacol.

[160] Harcherik DF, Leckman JF, Detlor J, Cohen DJ (1984). A new instrument for clinical studies of Tourette's syndrome. J Am Acad Child Psychiatry.

[161] Ricketts EJ, McGuire JF, Chang S, Bose D, Rasch MM, Woods DW (2018). Benchmarking treatment response in Tourette's disorder: a psychometric evaluation and signal detection analysis of the parent tic questionnaire. Behav Ther.

[162] Mårland C, Lichtenstein P, Degl’Innocenti A, Larson T, Råstam M, Anckarsäter H (2017). The Autism-Tics, ADHD and other comorbidities inventory (A-TAC): previous and predictive validity. BMC Psychiatry.

[163] McGuire JF, Piacentini J, Storch EA, Murphy TK, Ricketts EJ, Woods DW (2018). A multicenter examination and strategic revisions of the Yale Global Tic Severity Scale. Neurology.

[164] Haas M, Jakubovski E, Fremer C, Dietrich A, Hoekstra PJ, Jäger B (2021). Yale Global Tic Severity Scale (YGTSS): psychometric quality of the gold standard for tic assessment based on the large-scale EMTICS study. Front. Psychiatry.

[165] Abramovitch A, Reese H, Woods DW, Peterson A, Deckersbach T, Piacentini J (2015). Psychometric properties of a self-report instrument for the assessment of tic severity in adults with tic disorders. Behav Ther.

[166] Shaffer D, Gould MS, Brasic J, Ambrosini P, Fisher P, Bird H (1983). A children's global assessment scale (CGAS). Arch Gen Psychiatry.

[167] Cavanna AE, Schrag A, Morley D, Orth M, Robertson MM, Joyce E (2008). The Gilles de la Tourette syndrome-quality of life scale (GTS-QOL): development and validation. Neurology.

[168] Cloes KI, Barfell KS, Horn PS, Wu SW, Jacobson SE, Hart KJ (2017). Preliminary evaluation of child self-rating using the Child Tourette Syndrome Impairment Scale. Dev Med Child Neurol.

[169] Barfell KSF, Snyder RR, Isaacs-Cloes KM, Garris JF, Roeckner AR, Horn PS (2017). Parent and patient perceptions of functional impairment due to Tourette syndrome: development of a shortened version of the child Tourette syndrome impairment scale. J Child Neurol.

[170] Su MT, McFarlane F, Cavanna AE, Termine C, Murray I, Heidemeyer L (2017). The english version of the Gilles de la Tourette syndrome-quality of life scale for children and adolescents (C&A-GTS-QOL). J Child Neurol.

[171] Brazier J, Jones N, Kind P (1993). Testing the validity of the Euroqol and comparing it with the SF-36 health survey questionnaire. Qual Life Res.

[172] Balestroni G, Bertolotti G (2012). EuroQol-5D (EQ-5D): an instrument for measuring quality of life. Monaldi Arch Chest Dis.

[173] Cohen M (1988). The Revised Conners Parent Rating Scale: factor structure replication with a diversified clinical sample. J Abnorm Child Psychol.

[174] Bean J. Rey Auditory Verbal Learning Test, Rey AVLT. p 2174–2175 (2011)

[175] Engel-Yeger B, Josman N, Rosenblum S (2009). Behavioural Assessment of the Dysexecutive Syndrome for Children (BADS-C): an examination of construct validity. Neuropsychol Rehabil.

[176] Roth C (2011) Boston diagnostic aphasia examination. In: Kreutzer JS, DeLuca J, Caplan B (eds) Encyclopedia of clinical neuropsychology. Springer, New York, pp 428–430

[177] Spencer RJ, Wendell CR, Giggey PP, Seliger SL, Katzel LI, Waldstein SR (2013). Judgment of line orientation: an examination of eight short forms. J Clin Exp Neuropsychol.

[178] Roth C (2011) Boston naming test. In: Kreutzer JS, DeLuca J, Caplan B (eds) Encyclopedia of clinical neuropsychology. Springer, New York, pp 430–433

[179] Mahone EM, Cirino PT, Cutting LE, Cerrone PM, Hagelthorn KM, Hiemenz JR (2002). Validity of the behavior rating inventory of executive function in children with ADHD and/or Tourette syndrome. Arch Clin Neuropsychol.

[180] Rabin LA, Roth RM, Isquith PK, Wishart HA, Nutter-Upham KE, Pare N (2006). Self- and informant reports of executive function on the BRIEF-A in MCI and older adults with cognitive complaints. Arch Clin Neuropsychol.

[181] Homack S, Riccio CA (2006). Conners’ Continuous Performance Test (2nd ed.; CCPT-II). J Atten Disord.

[182] Homack S, Lee D, Riccio CA (2005). Test review: Delis–Kaplan executive function system. J Clin Exp Neuropsychol.

[183] Young AW, Rowland D, Calder AJ, Etcoff NL, Seth A, Perrett DI (1997). Facial expression megamix: tests of dimensional and category accounts of emotion recognition. Cognition.

[184] Nosek BA, Banaji MR (2001). The Go/No-Go association task. Soc Cogn.

[185] Tiffin J, Asher EJ (1948). The Purdue Pegboard: norms and studies of reliability and validity. J Appl Psychol.

[186] Van der Elst W, van Boxtel MP, van Breukelen GJ, Jolles J (2005). Rey's verbal learning test: normative data for 1855 healthy participants aged 24–81 years and the influence of age, sex, education, and mode of presentation. J Int Neuropsychol Soc.

[187] Meyers JE, Meyers KR (1995). Rey Complex Figure Test under four different administration procedures. Clin Neuropsychol.

[188] Manly T, Anderson V, Nimmo-Smith I, Turner A, Watson P, Robertson IH (2001). The differential assessment of children's attention: the Test of Everyday Attention for Children (TEA-Ch), normative sample and ADHD performance. J Child Psychol Psychiatry.

[189] Hélène V, Torny F, Druet-Cabanac A, Couratier P (2008). Use of the Visual Object and Space Perception (VOSP) test battery in two cases of posterior cortical atrophy. Neurocase.

[190] Spencer TD, Kruse L (2013) Beery-Buktenica developmental test of visual-motor integration. In: Volkmar FR (ed) Encyclopedia of autism spectrum disorders. Springer, New York, pp 400–404

[191] Valentine T, Block C, Eversole K, Boxley L, Dawson E (2020) Wechsler Adult Intelligence Scale-IV (WAIS-IV). The Wiley Encyclopedia of Personality and Individual Differences, pp 457–463

[192] McGrath MC (2011) Wisconsin card sorting test. In: Goldstein S, Naglieri JA (eds) Encyclopedia of child behavior and development. Springer US, Boston, pp 1571–1572

[193] Warschausky S (2011) Wechsler preschool and primary scale of intelligence. In: Kreutzer JS, DeLuca J, Caplan B (eds) Encyclopedia of clinical neuropsychology. Springer, New York, pp 2690–2693

[194] Watkins MW, Beaujean AA (2014). Bifactor structure of the Wechsler Preschool and Primary Scale of Intelligence-Fourth Edition. Sch Psychol Q.

[195] Wechsler D (1949) Wechsler intelligence scale for children; manual*.* The Psychological Corp., Oxford

[196] Elwood RW (1991). The Wechsler Memory Scale-Revised: psychometric characteristics and clinical application. Neuropsychol Rev.

